# What we learned from bench to bedside with the neuroblastoma targeting CD171-specific CAR

**DOI:** 10.1186/2194-7791-2-S1-A22

**Published:** 2015-07-01

**Authors:** A Künkele, AJ Johnson, C Berger, L Finn, J Park, MC Jensen

**Affiliations:** Pediatric Oncology and Hematology, Charité, Berlin, Germany; Fred Hutchinson Cancer Research Center, Seattle, WA USA; Pediatric Oncology and Hematology, Seattle Children's Hospital, Seattle, WA USA; Ben Towne Center, Seattle Children's Research Institute, Seattle, WA USA

## Meeting abstract

Despite the therapeutic efficacy of chimeric antigen receptor (CAR) redirected T cell immunotherapy in leukemia and lymphoma patients, similar clinical responses in solid tumor patients is, to date, an unrealized objective. We developed a CAR specific for CD171, an antigen expressed in several solid tumors including neuroblastoma, the most common extracranial tumor in childhood with an overall survival of less than 50% in high-risk patients. Since CD171 is also expressed in healthy tissues, including cerebellum and kidney, we proved the safety of targeting CD171 with CAR T cells in a non-human primate study. Further, we showed that CAR extracellular spacer and cytoplasmic signaling domain variants can be combined to tune the magnitude of cytotoxic CD8^+^ T lymphocyte (CTL) activation for tumor cell cytolysis and cytokine secretion. CAR constructs displaying the highest *in vitro* activity unexpectedly displayed the lowest *in vivo* anti-tumor activity, whereas CARs tuned for moderate signaling potency mediated tumor eradication. Recursively triggering hyperactive CARs rendered CTLs highly susceptible to activation-induced cell death resulting from augmented FasL expression, indicating that activation-induced cell death may be a critical parameter for achieving clinical efficacy against solid tumors. Our preclinical results assisted the design of a clinical trial comparing two CARs with different cytoplasmic signaling domains in patients with primary refractory or relapsed neuroblastoma, which was launched October 2014 at the Seattle Children's Hospital.

